# When Gynecological Anatomy Becomes a Surgical Emergency: A Rare Internal Hernia Case

**DOI:** 10.7759/cureus.87766

**Published:** 2025-07-12

**Authors:** Kirushanth Sathiyanathan, Nissanthan Tharmakulasingham, Senuri Dias, Chrishan Navarathnam, Eranga Perera

**Affiliations:** 1 Department of Surgery, Postgraduate Institute of Medicine, University of Colombo, Colombo, LKA; 2 Emergency Department, District General Hospital Avissawella, Avissawella, LKA; 3 General Surgery Department, District General Hospital Gampaha, Gampaha, LKA

**Keywords:** bowel ischemia, broad ligament defect, case report, internal hernia, small bowel obstruction

## Abstract

Internal hernias represent an uncommon etiology of small bowel obstruction (SBO). Among these, herniation through a defect in the broad ligament is one of the least common types. Because symptoms are vague and imaging findings are often minimal, these presentations can be easily overlooked and may progress to bowel ischemia or gangrene. A 47-year-old multiparous woman with no history of abdominal surgery presented to the emergency department with severe, worsening abdominal pain that developed over the past 12 hours. Ultrasound was the only imaging modality available. It detected free fluid in the abdomen but did not identify a specific organ abnormality. The clinical team initially proceeded with an open appendicectomy, which was subsequently converted to an exploratory laparotomy. During the procedure, they identified an internal hernia that had passed through a defect in the left broad ligament. Approximately 25 cm of the necrotic bowel was resected, and the remaining segments were joined with a primary anastomosis. Postoperatively, the patient recovered without incident and was discharged home once her bowel function resumed. While broad ligament hernias are exceedingly rare, they should be kept in mind as a possible explanation for obstruction in females who have not undergone pelvic surgery. Preoperative diagnosis is frequently missed because of subtle imaging clues and the infrequency of the disease in everyday practice. When advanced radiology is limited or inconclusive, surgical exploration remains the most reliable route to both identify the problem and provide timely treatment. Although rare, broad ligament hernias should be considered in female patients presenting with SBO and no prior abdominal operations. Prompt recognition and surgical intervention are vital to minimize morbidity and mortality.

## Introduction

Internal hernias are identified as a viscus protruding through a peritoneal or mesenteric opening in the abdominal cavity. Though rare, they can lead to small bowel obstruction (SBO) in 0.6% to 5.8% of cases [1]. Broad ligament hernias, while a subtype of internal hernias, are also a seldom identified pathology and account for 4% to 7% of internal hernias [2]. The broad ligament is a peritoneal fold attaching the fallopian tubes, ovaries, and uterus to the walls and floor of the pelvis [3]. Broad ligament hernias occur when loops of small bowel herniate through a defect in the broad ligament of the uterus.

Defects in broad ligaments can be congenital due to embryological defects or acquired due to trauma, pelvic inflammatory disease (PID), delivery of a child, or prior pelvic surgical procedures [3,4]. Congenital defects of the broad ligament are usually bilateral and thought to result from the spontaneous rupture of congenital cystic structures within it or a development abnormality of the broad ligament [1]. Defects can be classified as fenestrated (complete) or pouch-like (incomplete) [3]. The former allows for the bowel to pass freely, while the latter allows for loops of the bowel to become entrapped in a peritoneal recess.

Preoperative diagnoses are challenging because the clinical symptoms are nonspecific, such as nausea, vomiting, and abdominal pain, which makes it difficult to distinguish from other types of SBO, including adhesions, volvulus, or appendicitis [5]. Although computed tomography (CT) imaging may provide clues such as mechanical SBO with a double transition zone in the pelvis, dilated small bowel loops located laterally to the uterus, and increased separation between the uterus and one ovary, these findings are not specific [6]. CT imaging may aid in suspicion but often cannot definitively diagnose a broad ligament hernia. Surgical exploration remains the mainstay of definitive diagnosis and corrective surgery.

This case underscores the importance of early recognition of a broad ligament hernia, highlighting the challenges in preoperative diagnosis and the risk of ischemia to the herniated contents.

## Case presentation

A 47-year-old previously healthy multiparous woman presented to the peripheral surgical unit with acute generalized abdominal pain. She had no history of prior abdominal surgeries, trauma, gynecological conditions, or chronic medical illnesses.

The patient experienced generalized abdominal pain for one day, which was initially mild. She denied fever, urinary symptoms, or alterations in bowel habits and had a normal bowel opening with only mild abdominal distension. Upon initial examination, her abdomen was soft, with mild distension and no significant tenderness. On day 2, her condition deteriorated, with increasing abdominal distension, severe pain, nausea, and localized tenderness in the right iliac fossa (RIF), although she remained hemodynamically stable.

Laboratory investigations, including white blood cell (WBC) count and C-reactive protein (CRP), were repeatedly performed, which remained within normal limits (Table 1). An ultrasound scan of the abdomen revealed free fluid in the RIF and pouch of Douglas but did not show any significant abnormality suggestive of an underlying pathology. Due to the unavailability of CT imaging in the peripheral unit, further preoperative imaging could not be performed.

**Table 1:** Results of the laboratory investigations

Parameter	Measured Value on Day 1	Measured Value on Day 2	Reference Value
White blood cell count (x10^3^μL)	7	9	4.0-10.0
Neutrophil (%)	60	72	40-80
Hemoglobin (g/dL)	12.5	12.2	12.0-15.5
C-reactive protein (mg/L)	6	9.8	<10

**Figure 1:**
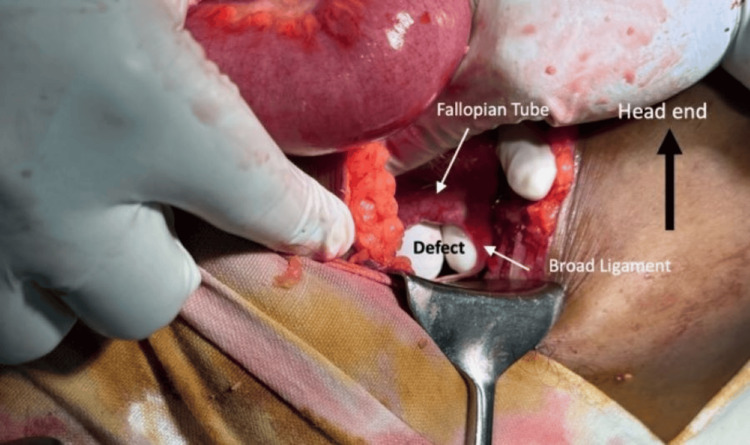
Perioperative findings of a 4-cm defect in the left broad ligament, which allowed ileal loops to herniate through it

On day 3, a clinical diagnosis of acute appendicitis was made in view of worsening RIF tenderness and persistent pain despite the absence of guarding and rebound tenderness. The patient was taken for emergency surgery. The procedure was initially intended to be an open appendicectomy, and a gridiron incision was made. Upon entering the peritoneal cavity, hemorrhagic fluid was encountered and sent for culture and sensitivity testing. The appendix appeared normal, but intraoperative findings revealed dilated small bowel loops. Consequently, the incision was extended medially to facilitate an exploratory laparotomy. Further exploration revealed an internal herniation of ileal loops through a 4-cm defect in the left broad ligament (Figure 1).

The herniated bowel loops were carefully reduced, and the viability of the strangulated segment was assessed. Due to persistent discoloration, absent peristalsis, and uncertain viability, resection of the compromised 25-cm bowel segment was performed, followed by an end-to-end anastomosis. To prevent future herniation, the broad ligament defect was closed.

Postoperatively, the patient had an uneventful recovery with gradual improvement in bowel function. The abdominal drain was removed after confirming the absence of significant fluid collection.

## Discussion

Internal herniation through the broad ligament is an uncommon but potentially life-threatening condition causing SBO. If left unaddressed, it may lead to catastrophic outcomes. In this patient, a lack of history of previous surgeries or adhesions predisposed other causes of SBO, stressing the value of keeping a broad differential diagnosis.

**Figure 2:**
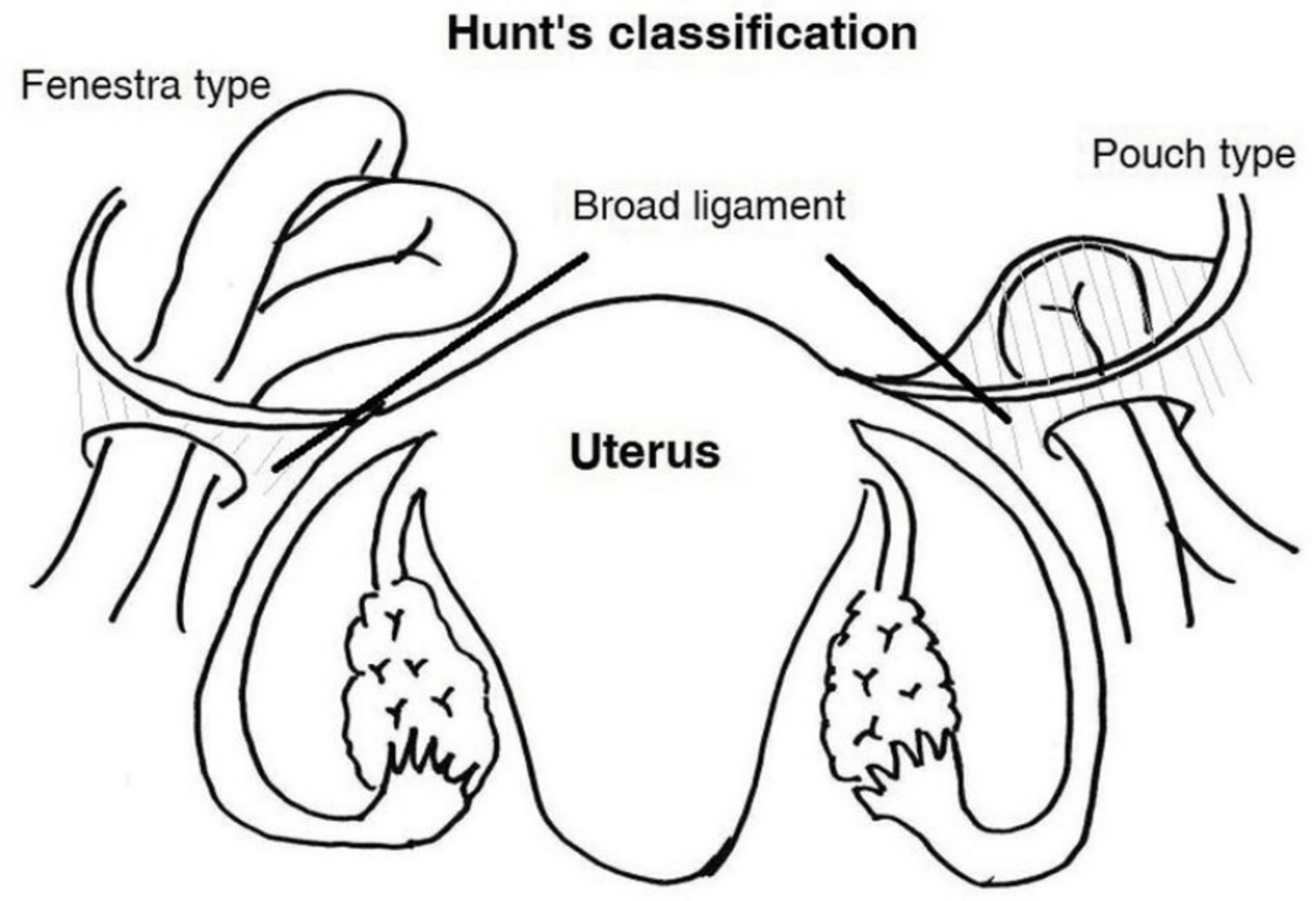
Schematic diagram showing the classification of broad ligament hernia: Hunt’s classification divides the disease into fenestra and pouch types on the basis of the nature of the hernia defects Source: [7]

As shown in Figure 2, broad ligament hernias are caused by peritoneal layer defects of the ligament. Fenestrated is the most common variety, where bowel loops migrate through a full-thickness defect [7]. Congenital ones occur as a result of imperforate fusion, disturbed Müllerian duct development, or spontaneous rupture of congenital cystic structures within the broad ligament. Acquired defects are caused by obstetric trauma, pelvic operation, or chronic inflammatory diseases such as PID [3,4]. Increasing intra-abdominal pressure from pregnancy, exercise, or distension of the bowel can lead to herniation in pregnant females with pre-existing ligamentous broad ligament weakness.

Although the ileum is most commonly involved in broad ligament hernias, other structures such as the fallopian tubes may also become incarcerated, as shown in previous studies [1,4].

The majority of cases are diagnosed intraoperatively [8]. Preoperative diagnosis is challenging. Clinical presentation is often that of appendicitis, gynecological emergencies, or a general SBO. The lack of availability of CT imaging, as seen in this case, is one factor that creates uncertainty.

If they are available, contrast CT will show dilated loops of small bowel near the uterus or fallopian tubes, typically with a point of apparent transition in the pelvis [2]. However, despite sophisticated imaging, hernia may not be correctly demonstrated; therefore, early surgical investigation in those patients in whom the condition is worsening is extremely important.

Surgical management involves reduction of the herniated bowel, examination of its viability, resection of any nonviable segment, and closure of the defect. The choice between open repair and laparoscopy remains controversial. However, laparoscopic surgery is contraindicated when the small bowel diameter exceeds 4 cm, there is evidence of ischemia, there is a history of severe adhesions, or there is a presence of inflammatory bowel disease [2]. In stable patients without ischemia, laparoscopic repair has proved to be effective and is associated with a shorter recovery time and less postoperative pain [3]. In hemodynamically unstable patients in whom bowel necrosis is suspected, an open approach remains the best method. In addition, a second-look laparotomy within 24 to 48 hours of the initial surgery may be considered in certain patients with questionable bowel viability [9]. Table 2 presents a summary of results of a literature review of similar studies [1-4].

**Table 2:** Review of recent studies reporting broad ligament hernia Source: [1-4]

Reference	Takahashi et al. [1]	Wang et al. [2]	R et al. [3]	Rohatgi et al. [4]	Rohatgi et al. [4]
Age	52 years	68 years	88 years	35 years	42 years
Presentation	Lower abdominal pain and vomiting	Lower abdominal pain	Abdominal pain, constipation, bilious vomiting	Abdominal pain, vomiting, constipation	Abdominal pain, bilious vomiting, constipation
Risk factors	History of bowel obstruction following cesarean section	Multiparity	Multiparity	Recent cesarean section	None
Surgery	Laparoscopy converted to laparotomy	Exploratory laparotomy	Exploratory laparotomy	Laparoscopy	Laparoscopy
Management	Strangulated ileal loop preserved as no ischaemic changes. Right-sided salpingectomy performed due to gangrenous ampulla of right fallopian tube.	Nonviable small bowel resected and end-to-end anastomosis performed.	Preservation of the small bowel due to viability. Closure of broad ligament defect.	Preservation of the small bowel due to viability. Salpingectomy performed due to gangrenous fallopian tube.	Closure of broad ligament defect with preservation of bowel loops since hernial contents had spontaneously reduced
Postoperative recovery/recurrence	Uneventful/No	Uneventful/No	Uneventful/No	Uneventful/No	Uneventful/No

Diagnosis of intraoperative broad ligament hernias includes gentle probing. It is especially relevant in women with SBO and no surgical history. Repair of the defect should be performed to prevent recurrence.

This case highlights the need to retain a broad differential frame of mind for SBO presentations, consider internal hernias even in patients with no surgical history, and act quickly on the basis of clinical judgment, especially in resource-limited settings.

It is important to act quickly since delays in acting firmly may result in bowel infarction, sepsis, and death. Clinicians must remain extremely cautious in multiparous women due to repeated mechanical stress that may increase the risk of defects in ligaments.

## Conclusions

Broad ligament hernias are an important cause of SBO in women, even though they are rare. Diagnosis is often made intraoperatively due to their non-specific presentation and difficulty in radiology detection. It is of paramount importance to have high clinical suspicion and perform timely surgical intervention to reduce the associated morbidity and mortality. Our case illustrates the critical role of surgical exploration in resource-limited settings and underscores the need to consider internal hernias in the differential diagnosis of SBO, especially in women without typical risk factors such as prior abdominal surgery or trauma.
